# Mönckeberg's Disease With Calcified Lower Limb Ischemia in Saudi Arabia: A Rare Case Report and Literature Review

**DOI:** 10.7759/cureus.38345

**Published:** 2023-04-30

**Authors:** Ahmed M Odah, Mohammed O Khalid, Ahmed A Alsaati, Hawra A Alqassab, Ghadeer R Alkouder, Murtadah H Alhejji, Jafar A Alkathem, Abdulsalam Aleid

**Affiliations:** 1 General Surgery, King Faisal University, Al Ahsa, SAU; 2 General Surgery, Prince Saud Bin Jalawy Hospital, Al Ahsa, SAU; 3 Nurse Wound Care, Prince Saud Bin Jalawy Hospital, Al Ahsa, SAU; 4 Radiological Sciences, King Saud Bin Abdulaziz University for Health Sciences, Al Ahsa, SAU; 5 Internal Medicine, King Faisal University, Al Ahsa, SAU

**Keywords:** mönckeberg's, general surgery, vascular surgery, lower limb ischemia, calcified

## Abstract

Mönckeberg's disease, a rare medial calcific arteriosclerosis, predominantly affects lower extremity arteries with an unclear etiology. If untreated, severe complications like ischemic necrosis and gangrene may arise. We present a case of a 28-year-old male with spontaneous lower limb swelling, abscess, and itching. Despite a history of deep vein thrombosis and warfarin therapy, Mönckeberg's disease was suspected. Imaging revealed diffuse vascular calcification of the media of the arterial wall bilaterally in the right and left femoral vessels with heterogenous irregular soft tissue collection in the right with suspected infection. Following treatment, the patient's symptoms improved, and follow-up imaging showed resolution of fluid collections and improved calcification appearance. This report highlights the importance of considering Mönckeberg's disease in the differential diagnosis of lower extremity swelling and the need for timely management to prevent serious complications.

## Introduction

Ischemia is caused by inadequate circulation to the legs and feet. The most prevalent cause [1] is atherosclerotic peripheral arterial disease, characterized by atheromatous plaques in the arteries of the lower extremities. Risk factors for atherosclerosis include family history, male gender, age, tobacco use, diabetes, dyslipidemia, and hypertension [2]. Severe lower extremity ischemia can occur in the absence of these risk factors. Rare causes and atypical atherosclerosis must be considered in these patients [3].

In this case, ischemia of the left lower limb was caused by Mönckeberg's arteriosclerosis, which causes diffuse calcification of the tunica media of medium-sized arteries. The patient was a 28-year-old male with no chronic medical illness or surgical history and was fully vaccinated with three doses of the Pfizer COVID-19 vaccine. Mönckeberg's arteriosclerosis may be caused by genetics, endothelial damage, or disorders of calcium and phosphate metabolism. In men above 50 years, Mönckeberg's arteriosclerosis causes intermittent claudication or ischemic skin changes in the lower extremities. Mönckeberg's arteriosclerosis is a rare cause of limb ischemia that should be considered in patients without cardiovascular risk factors. For the most effective treatment of lower extremity ischemia, a prompt and accurate diagnosis is necessary.

Misdiagnosis or delay in diagnosing rare diseases such as Mönckeberg's arteriosclerosis can result in amputation or death. Even in the absence of peripheral arterial disease risk factors, this case demonstrates the importance of clinician awareness and a high index of suspicion for unusual causes of lower limb ischemia. A comprehensive medical history, physical examination, and diagnostic tests can assist in identifying rare causes. Although atherosclerotic peripheral arterial disease is the most common cause of lower limb ischemia, clinicians should also consider fewer common causes such as Mönckeberg's arteriosclerosis.

In patients devoid of atherosclerosis risk factors, careful diagnosis and the suspicion of rare diseases are required. This case report demonstrates an uncommon cause of limb ischemia, prompting clinicians to consider all potential etiologies in order to make an accurate diagnosis and administer the most effective treatment.

## Case presentation

**Figure 1:**
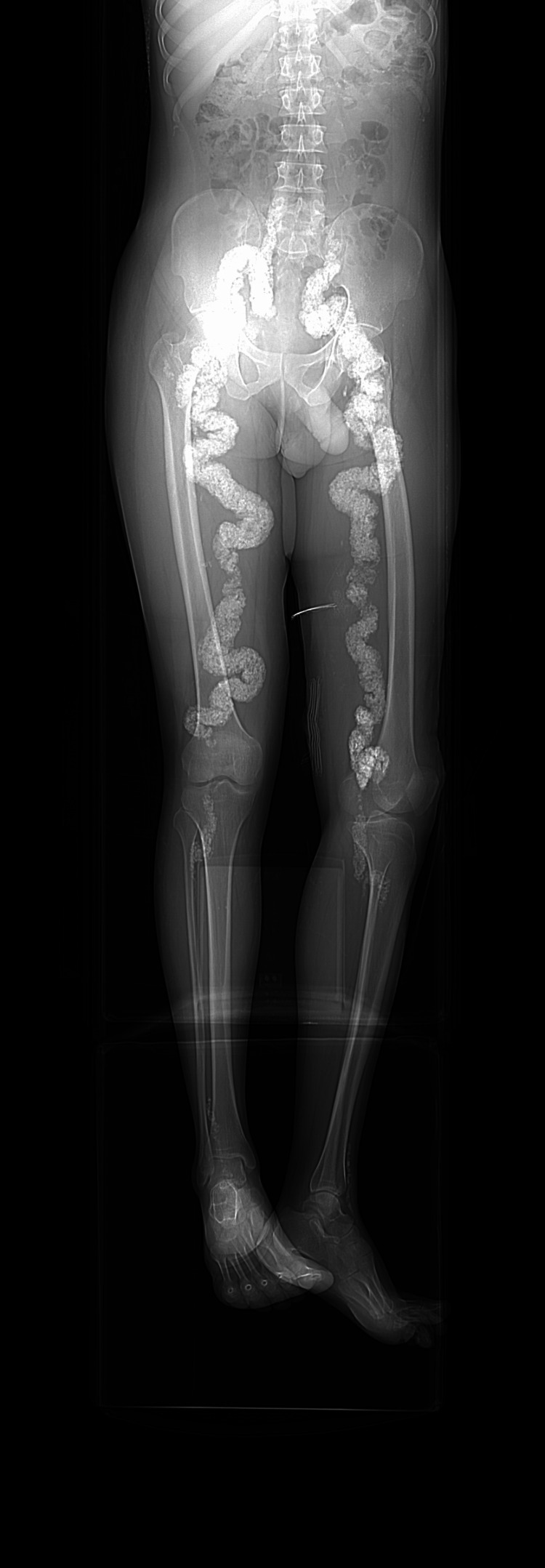
Plain radiograph of the femur, anteroposterior (AP) view The image shows a heavily calcified tortuous vascular structure, limiting the underlying bony assessment for clinical correlation. Scattered amorphous soft tissue calcification was noted.

**Figure 2:**
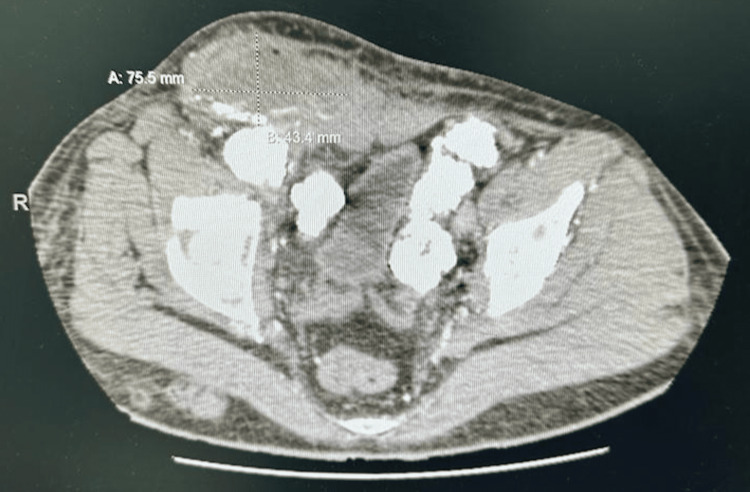
CT angiography of lower extremities The findings redemonstrated the diffuse extensive amorphous vascular/perivascular calcifications. Heterogenous irregular soft tissue collection seen in close contact with the right femoral artery and veins presently measured approximately 7.5 cm x 4.3 cm with minimal contrast extravasation. Few air foci were noted within, which could be due to superimposed infection. However, bilateral common iliac arteries and both posterior tibial arteries appeared normal. There was an inability to assess the latency of the rest of the lower limb arteries due to extensive wall calcifications.

We present a 28-year-old Indian male patient who was not known to have any chronic medical illness, trauma, or surgical history. He presented due to spontaneous bilateral swelling in both of his thighs with itching in his right thigh. He complained of severe pain related to the swelling, which never had similar episodes in the past. On examination, the patient was unable to walk normally, both of his feet were warm but with good capillary refills; however, his pulsations were bilaterally impalpable. There was softness with pitting edema in his right thigh and calf; on the other hand, an abscess collection was noticed in his left leg. Doppler examination revealed a biphasic sound in the right common femoral artery, while dorsal pulses were bilaterally absent. An ultrasound showed calcified arteries, but there was an inability to visualize the right femoral vein. The patient also reported a history of deep vein thrombosis (DVT) but was compliant with warfarin therapy. The workup for diagnosis comprised clinical history, physical examination findings, hematological investigations, and imaging studies of differential diagnosis including Mönckeberg's disease, atherosclerosis, and thromboangiitis obliterans. After taking all of the clinical information into account, Mönckeberg's disease was suspected. A plain radiograph of the femur confirmed heavily calcified tortuous vascular structures and soft tissue calcification, supporting the diagnosis. Initially, plain radiograph of the femur, anteroposterior (AP) and lateral views, showed heavily calcified tortuous vascular structure, limiting the underlying bony assessment for clinical correlation; additionally, scattered amorphous soft tissue calcification was noted (Figures 1, 2).

The patient underwent a multiplanar, multisequential magnetic resonance imaging (MRI) of the pelvis with contrast, which showed diffuse vascular calcification and a large, irregular, heterogeneous soft tissue collection in close contact with the right femoral artery and veins. Furthermore, there was a wall disruption of the right femoral artery with connection to this mass, measured approximately 8.7 cm x 7.1 cm x 6.5 cm in its maximum dimensions. The post-contrast images showed diffuse thick peripheral wall enhancement as well as edema and enhancement surrounding the mass. Moreover, there were multiple fluid collections in the right thigh, which showed a high signal on T2 and a low signal on T1 with peripheral contrast enhancement; however, no abnormal bone marrow signal changes were noted. A computed tomography (CT) angiography of the lower extremities was done later to compare the findings with those shown within the MRI, which showed diffuse extensive amorphous vascular calcifications and heterogenous irregular soft tissue collection in close contact with the right femoral artery and veins (Figure 2).

**Figure 3:**
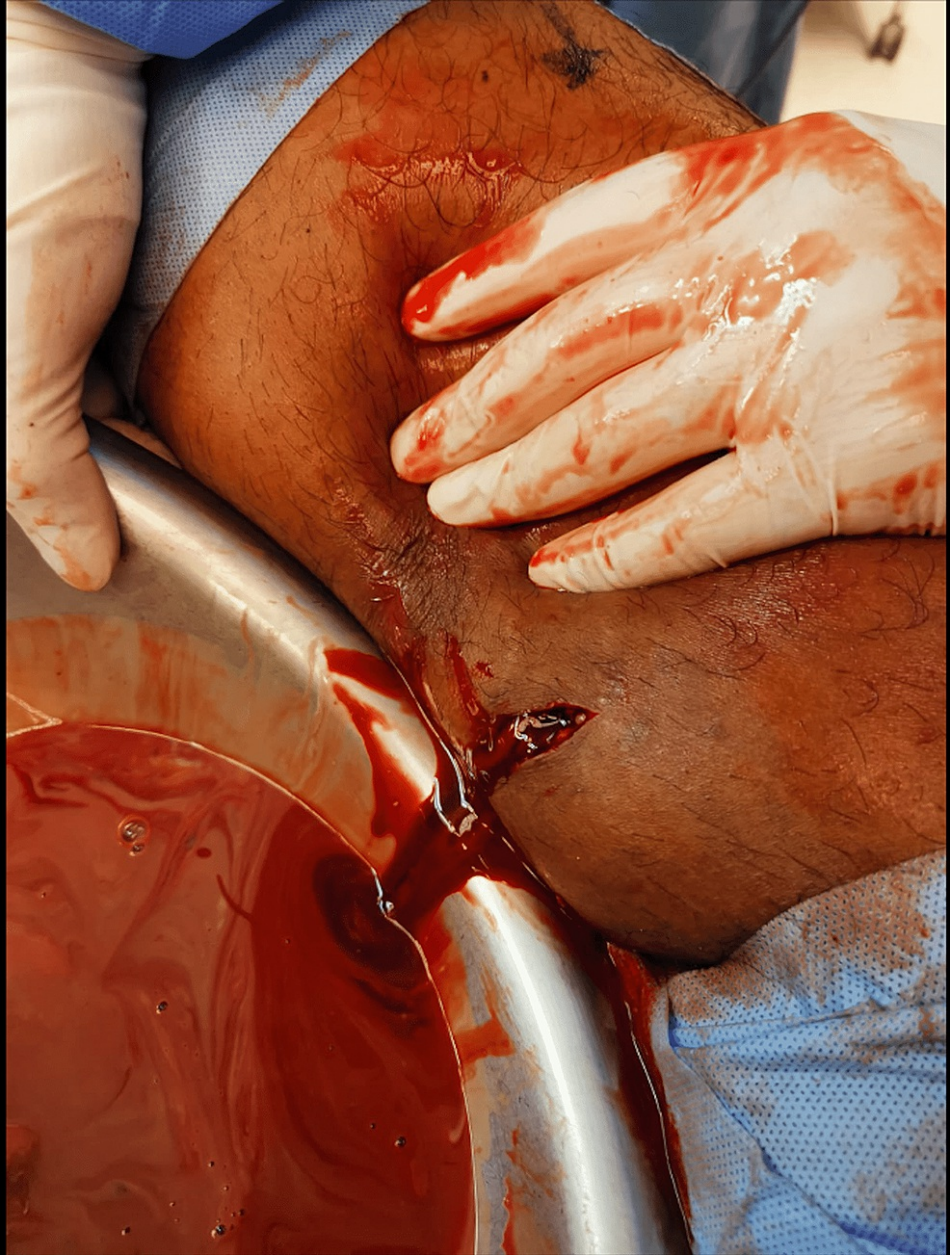
Gross image of the fluid collections located in the right thigh

The patient was started on intravenous antibiotics, ceftriaxone 2 g daily and vancomycin 1 g every 12 hours, for the suspected infection in the left leg. As for the hematoma in the right femoral vessel, the patient was initially managed with conservative measures, including bed rest and ice packs, along with anticoagulant therapy with warfarin 5 mg daily to prevent any further clot formation. The patient's international normalized ratio (INR) was maintained between 2 and 3. Following treatment, the patient's symptoms improved, but he was not able to walk without interventional surgery to drain the fluid collection from the left thigh as shown (Figure 3). The patient’s symptoms have improved with the treatment as he was able to walk without any difficulties.

Afterward, a follow-up imaging was done, which showed resolution of the fluids’ collections and improvement of the appearance of the arterial calcification.

Conclusively, the findings described above were highly suggestive of injury to the right femoral vessel, with a superimposed hematoma and infection. Multiple fluid collections were present in the right thigh (Figure 3).

While atherosclerotic disease, characterized by the formation of atheromatous plaques within arteries, is the primary cause of lower limb ischemia [3], this condition is usually associated with major cardiovascular risk factors, including family history, male sex, older age, smoking, diabetes mellitus, dyslipidemia, and hypertension [4]. However, when severe ischemia presents in the absence of these risk factors, alternative etiologies and pathologies should be considered [5]. We report an exceptional case of critical lower limb ischemia resulting from Mönckeberg's arteriosclerosis.

Mönckeberg's arteriosclerosis does not preclude revascularization in all cases. In some situations, anastomoses are possible without clamping the artery. However, in our case, the pathology was so diffuse, thickened, and calcified that it prevented clamping and standard arterial revascularization, necessitating primary amputation [4]. Shabat et al. reported a case of popliteal artery rupture during orthopedic knee surgery due to intense arterial wall calcification [6].

In patients with peripheral obstructive arterial disease of undefined etiology lacking traditional risk factors, Mönckeberg's medial layer calcification, though rare, should be considered a potential cause of peripheral vasculopathy. Further studies are needed to better understand this disease and improve diagnosis and treatment [7].

## Discussion

Mönckeberg's medial layer calcification, though rare, should be considered in patients with peripheral obstructive arterial disease of unknown etiology and no conventional risk factors [7]. Research is needed to improve the diagnosis and treatment of this disease [1]. According to Ho et al., type 2 diabetes is rising [8]. This condition increases the risk of microangiopathies and macroangiopathies [9], including arterial media calcification-associated Mönckeberg's sclerosis [10]. This review discusses the history of Mönckeberg's sclerosis, morphological forms, etiopathogenesis, recent findings on medial arterial calcification's effects on hemodynamics and cardiovascular risks, and diagnostic methods [5]. Vascular calcification has been studied for centuries due to its links to aging, atherosclerosis, and cardiovascular disease. The mechanisms of medial arterial calcification (MAC) in multifactorial peripheral arterial disease (PAD) are unknown. Surgery induces limb ischemia in PAD animal models. VSMC stands for vascular smooth muscle cell, which is a type of muscle cell that is found in the walls of blood vessels. These cells have a crucial role in regulating blood pressure, blood flow, and vascular tone [8]. VSMCs drive vascular calcification in aging, diabetes, and chronic kidney disease, which often co-occur with PAD and worsen its symptoms. Calcified/aged VSMCs may rupture plaque directly and indirectly [5].

Nonetheless, a few questions remain as follows: How much persistent DNA damage from oxidative stress or other factors does PAD cause? Is oxidative stress a cause of diabetic neuropathy? Is there Prelamin A accumulation in PAD? Neurotrophins affect VSMC phenotypes. Understanding risk factor interactions, modulating the VSMC phenotype, and more sensitive detection of PAD and MAC (e.g., 18F-sodium fluoride PET/CT, 18F-FDG PET/CT) could improve management [5]. PAD is linked to atherosclerosis and MAC. Aging increases both risks. Oxidative stress from diabetes and chronic kidney disease damages cells and nuclear lamina, accelerating VSMC senescence. Osteogenic VSMCs' SASP stands for senescence-associated secretory phenotype, which refers to a phenomenon in which senescent cells, including VSMCs, secrete various pro-inflammatory cytokines, chemokines, growth factors, and other factors. This can promote chronic inflammation and tissue damage and contribute to the development and progression of age-related diseases, including atherosclerosis and PAD [8]. SASP may help atherosclerotic plaques progress and rupture, causing peripheral artery occlusion [5]. Peripheral neuropathy increases MAC and PAD risk. Atherosclerotic disease, which causes atheromatous plaques in arteries, is the leading cause of lower limb ischemia [3]. Major cardiovascular risk factors include family history, male sex, older age, smoking, diabetes, dyslipidemia, and hypertension [4]. Without these risk factors, alternative etiologies and pathologies [5] should be considered for severe ischemia. Revascularization can be done with Mönckeberg's arteriosclerosis (Table 1). An anastomosis can be done without clamping the artery. Our pathology was so diffuse, thickened, and calcified that clamping and standard arterial revascularization failed, necessitating primary amputation [4]. Due to intense arterial wall calcification, Shabat et al. [6] reported popliteal artery rupture during orthopedic knee surgery. Mönckeberg's medial layer calcification, though rare, may cause peripheral vasculopathy in patients with peripheral obstructive arterial disease of unknown etiology and no conventional risk factors. More research is needed to understand and treat this disease [7].

**Table 1:** Review of literature on some of the documented Mönckeberg's diseases

First author (Year)	Study type	No. of cases	Sex	Age	Comorbidities	Chief complaints, findings, and diagnosis	Management
Top et al. (2002) [10]	Case report	1	Male	20 years old	Diabetes mellitus and chronic renal failure	Extensive peripheral artery calcification, medial artery calcification in temporal artery biopsy. Suggested unusual form of Mönckeberg’s sclerosis.	NA
Naha et al. (2012) [11]	Case report	1	Male	62 years old	Diabetes mellitus type II	Chief Complaint: Angina at rest for the last 6 days. Findings: Calcification in digital arteries of both hands in plain X-ray. Left anterior descending artery with extensive proximal adventitious calcifications with a mid-95% discrete stenosis. Left circumflex coronary artery had proximal 90% stenosis followed by total occlusion. Findings were consistent with Mönckeberg’s sclerosis involving bilateral radial and ulnar arteries.	Percutaneous angioplasty with drug-eluting stents to the right coronary artery and left anterior descending artery in a staged approach. Statin and antiplatelets were also given.
Phelps et al. (2015) [12]	Case report	1	NA	NA	History of acute vision loss without other symptoms of temporal arteritis	Chief complaint: Acute vision loss. Findings: Hard artery to palpation. Mineralization of internal elastic laminate, consistent with Mönckeberg’s arteriosclerosis.	NA
Coronado et al. (2017) [13]	Case report	1	Female	46 years old	Arterial hypertension, chronic kidney disease stage 5, and type II diabetes mellitus	Dystrophic calcification associated with changes compatible with Mönckeberg’s disease	Infracondylar amputation of the left lower limb
Castillo et al. (2020) [14]	Case reports	2	Case 1: Female; Case 2: Male	Case 1: 69 years old; Case 2: 67 years old	Case 1: Hypertension, type II diabetes mellitus, and chronic headache; Case 2: Hypertension, asthma, hyperlipidemia, and cataract procedure history	Case 1: Chief complaint: Presented due to worsening bitemporal headaches. Findings: Left temporal artery biopsy revealed temporal artery calcification of tunica media, which is consistent with Mönckeberg’s medial calcific sclerosis. Case 2: Chief complaint: Presented with a blurry vision of the left eye with an intermittent headache. Findings: Left temporal artery biopsy revealed elastic fibers by calcification consistent with Mönckeberg’s medial calcific sclerosis.	Case 1: Prednisone 60 mg daily for one week. Case 2: Solumedrol 120 mg in an emergency. Prednisone 70 mg daily.
Belliveau et al. (2013) [15]	Case report	1	Female	85 years old	Osteoporosis, hypothyroidism, coronary artery disease, osteoarthritis, and primary lung neoplasm	Chief complaint: Left-sided scalp tenderness, jaw claudication, left-sided hearing loss. Findings: Light microscopic examination of the biopsy specimen (27 mm) showed no evidence of active or healed arteritis. However, there were prominent areas of calcification with focal ossification including osteoclast-type giant cells. The calcification was located in the tunica media and also present vocally at the internal elastic laminate. These findings are consistent with Mönckeberg’s disease.	Prednisone 60 mg/day for 10 days. Tapered over 2 weeks and treatment discontinued.
Chauhan et al. (2022) [16]	Case report	1	Male	56 years old	Diabetes mellitus type II	Chief complaint: Twisting of the left ankle for 1 day. Food X-ray revealed calcified posterior tibia artery and dorsal pedis artery (railroad track pattern calcification). The patient was diagnosed with soft tissue injury left ankle along with incidentally detected Mönckeberg’s sclerosis.	Was started on atorvastatin 40 mg/day, aspirin 75 mg/day along with oral hypoglycemic drugs, and referred to a cardiologist for further cardiovascular evaluation.
Odah et al. (Present case)	Case report	1	Male	28 years old	Medically free	Chief complaint: Spontaneous bilateral swelling of both legs with itching in his right leg. Ultrasound showed calcified arteries with an inability to visualize the right femoral vein. A plain radiograph of the femur showed a heavily calcified tortuous vascular structure with scattered amorphous soft tissue calcification. The diagnosis was concluded as Mönckeberg’s disease.	Was started on antibiotics and anticoagulant therapy for the suspected infection and hematoma, respectively. The patient’s symptoms have improved with the treatment as he was able to walk without any difficulties.

Acute limb ischemia (ALI) has high mortality and amputation rates according to Olinic et al. [9]. This review updated ALI diagnosis and treatment. This article covered all ALI causes, focusing on embolism and in situ thrombosis. Here, clinical data and imaging (particularly duplex ultrasound, CT angiography, and digital subtraction angiography) were stressed in emergency diagnosis. This article discussed pharmacological (thrombolysis), interventional (thromboaspiration, mechanical thrombectomy, and stent implantation), surgical (Fogarty thromboembolectomy, bypass, endarterectomy, patch angioplasty, or combinations), and minor or major amputation as well as modernized postoperative management, reperfusion injury, compartment syndrome, and long-term treatment [11]. According to the data, there were too few randomized studies on management strategies. New endovascular techniques are promising, but they are only used in specialized centers. Surgery, however, is common and beneficial. Recent ALI registry recommendations aim to improve things. Table 1 shows the rare case reports reported of Mönckeberg's disease.

Our 28-year-old patient had bilateral swelling in his right thigh and left thigh, heterogeneous irregular soft tissue collection seen in close contact with the right femoral artery, and itching in his right thigh. However, specialized imaging techniques such as CT or MRI may be able to detect the presence of calcium deposits in the arterial walls, which can help diagnose Mönckeberg's disease. In particular, high-resolution CT imaging with contrast has been shown to be a reliable method for detecting and quantifying arterial calcification in Mönckeberg's disease.

Imaging studies revealed Mönckeberg's disease. After treatment, his swelling went down and he could walk again. The fluid collection and arterial calcification improved on his follow-up imaging. Due to macroangiopathies, diabetes mellitus is strongly linked to Mönckeberg's sclerosis according to Kulikova et al. [7]. Our patient was medically healthy, unlike the previous study. In Ho et al.'s study [8], MAC and PAD were linked to vascular calcification by aging, atherosclerosis, and cardiovascular disease. The study also found that new imaging modalities can improve PAD and MAC detection sensitivity, supporting our CT angiography case study. Diffuse extensive amorphous vascular calcifications and heterogenous irregular soft tissue collection near the right femoral artery and vein were found. These findings improved patient management. According to Olinic et al.'s study [9], ALI can be treated with pharmacological (thrombolysis), interventional (thromboaspiration, mechanical thrombectomy, and stent implantation), surgical (Fogarty thromboembolectomy, bypass, endarterectomy, patch angioplasty, or combinations), or minor or major amputation [6]. In our case study, antibiotics and anticoagulant therapy for the suspected infection and hematoma saved the patient and allowed him to walk without intervention or amputation.

## Conclusions

This case report illustrates the importance of considering Mönckeberg's disease in the differential diagnosis of patients with swelling in the lower extremities, particularly those with risk factors such as advanced age, male gender, hypertension, diabetes mellitus, and hyperlipidemia. It also highlights the need for prompt diagnosis and treatment of the disease to prevent serious complications, such as ischemic necrosis and gangrene. The successful management of this patient's condition led to a favorable outcome, with improvement in his symptoms and resolution of the fluid collections.
